# Development of a Multi-Dimensional Parametric Model With Non-Pharmacological Policies for Predicting the COVID-19 Pandemic Casualties

**DOI:** 10.1109/ACCESS.2020.3044929

**Published:** 2020-12-15

**Authors:** Onder Tutsoy, Adem Polat, Şule Çolak, Kemal Balikci

**Affiliations:** 1 Department of Electrical-Electronics EngineeringAdana Alparslan Türkeş Science and Technology University 01250 Adana Turkey; 2 Department of Electrical and Electronics EngineeringOsmaniye Korkut Ata University 187481 80000 Osmaniye Turkey

**Keywords:** COVID-19 casualties, non-pharmacological approaches, pandemic, parametric model, prediction, SIR model, SpID model, SpID-N model

## Abstract

Coronavirus Disease 2019 (COVID-19) has spread the world resulting in detrimental effects on human health, lives, societies, and economies. The state authorities mostly take non-pharmacological actions against the outbreak since there are no confirmed vaccines or treatments yet. In this paper, we developed Suspicious-Infected-Death with Non-Pharmacological policies (SpID-N) model to analyze the properties of the COVID-19 casualties and also estimate the future behavior of the outbreak. We can state the key contributions of the paper with three folds. Firstly, we propose the SpID-N model covering the higher-order internal dynamics which cause the peaks in the casualties. Secondly, we parametrize the non-pharmacological policies such as the curfews on people with chronic disease, people age over 65, people age under 20, restrictions on the weekends and holidays, and closure of the schools and universities. Thirdly, we explicitly incorporate the internal and coupled dynamics of the model with these multi-dimensional non-pharmacological policies. The corresponding higher-order and strongly coupled model has utterly unknown parameters and we construct a batch type Least Square (LS) based optimization algorithm to learn these unknown parameters from the available data. The parametric model and the predicted future casualties are analyzed extensively.

## I. Introduction

An epidemic of cases with unexplained low respiratory infections are classified as “pneumonia of unknown etiology” detected in Wuhan, was first reported to the World Health Organization (WHO) country office in China on December 31, 2019. This new virus, whose etiology is attributed to the coronavirus (CoV) family, was named “COVID-19”, which stands for “coronavirus disease 2019” by the WHO [1]. CoVs are classified as alpha-(infected from bats), beta-(infected from bats), gamma-(infected by birds and pigs), and delta- (infected by birds and pigs) coronaviruses [2], [3]. In the past two decades, several viral outbreaks that pose a serious public health risk have been reported by the WHO. The Severe Acute Respiratory Syndrome (SARS) coronavirus from 2002 to 2003, Influenza A (H1N1) in 2009, and the Middle East Respiratory Syndrome (MERS) coronavirus in 2012 have been reported [1], [4]. The current COVID-19 outbreak has both similarities and differences when comparing to SARS and MERS outbreaks where they had both zoonotic transmission (SARS: likely from bats via palm civets, MERS: likely from bats via dromedary camels). All 3 viral infections usually occur with fever and cough causing the lower respiratory [5]. COVID-19 is considered a deadlier epidemic than SARS and MERS because it has affected more people over a period of time compared to the other two outbreaks (SARS; 8,437 cases, 813 deaths, 9.63% mortality rate. MERS; 2,499 cases, 861 deaths, 34.45% mortality rate, according to the report on March 3, 2020) [6], COVID19; 19,131,120 cases (increasing), 714,873 deaths (increasing), 3.73% mortality rate, by 8 August 2020) [7]).

In the early stages of an infectious outbreak, understanding the transmission dynamics of the infection can provide insights about its behavior and determine whether the outbreak control measures are yielding a significant impact on the casualties [8], [9]. Mathematical and statistical modelling is a valuable tool to comprehensively analyze the dynamics of infectious diseases, population behavior, the availability of public health resources, and the effectiveness of public health interventions (such as social distancing) [10], [11]. Such modelling based analysis can provide predictions about the future growth potential of the epidemic, guiding estimation of risk to other countries, and planning the alternative policies [12]–[14]. To develop models, training data (epidemiological, non-pharmacological, population, and travel) and model validation data are required where the resulting models can estimate the local and global spread of the infectious diseases [15]. There are several mathematical approaches to perform parametric modelling of the outbreaks (e.g. CoVs family: COVID-19, SARS, MERS) such as Susceptible-Infectious (SI), Susceptible-Infectious-Susceptible (SIS), Susceptible-Infectious-Recovered (SIR), which is one of the most widely considered mathematical models for predicting the spread of pandemics, and the variants of SIR are Susceptible-Infectious-Recovered-Susceptible (SIRS), and Susceptible-Exposed-Infectious-Recovered (SEIR) models [16]–[18].

SEIR model-based infection model characterization of pandemic disease is shown in Fig. S1 [18]. Ignoring the exposed people reduces the SEIR model to the SIR model. The SI model is the simplest form of all disease models and matches the behavior of diseases such as cytomegalovirus (CMV) or herpes. In this model, susceptible individuals remain infected and infectious throughout their lives and remain in contact with the susceptible population if they are infected and receive no treatment [18], [19]. The SIS model is suitable for infections recurring frequently, such as the common cold (rhinoviruses) or sexually transmitted diseases such as gonorrhea or chlamydia, where infected individuals return to a susceptible state after infection [18], [19]. SIRS representing the transmission of an infectious disease through individuals has 4 possibilities such as susceptible, infectious, recovered, and again being susceptible where susceptible is monitored and large epidemics affecting the world can be modelled more effectively compared to SI and SIS models. Recovery rate and the time scale of infection are the two key quantities that govern epidemic dynamics at the population level for SIS, SIR, and SEIR. The time scale of the infection is measured by the infectious period of SIS and SIR models or by a mixture of exposed and infectious periods of the disease with the SEIR model [18]. In the SIS model, individuals move from susceptible to infections and then return to susceptible upon recovery, and recovery does not provide immunity. If individuals recover and gain permanent immunity, the model becomes a SIR model. If individuals recover with transient immunity and eventually become susceptible again, the model becomes a SIRS model, whereas if individuals do not recover, the model is an SI model [20]. A study modeling the exposure of residential areas to epidemics by synthesizing climatic, environmental, demographic, and health risk factors with an Index c whose value ranges from 0 to 1 and defines the severity of transmission of the epidemic was proposed [21]. A stochastic transmission model parameterized to the COVID-19 was developed to investigate if the contact tracing and the isolation of cases could control the SARS-CoV-2 pandemic [22]. Kucharski, et.al., in their study, modeled the stochastic transmission dynamics of the infection as a geometric random walk process implementing COVID-19 data in a mathematical model SARS-CoV-2 transmission. They made inferences for transmission rate and for the numbers of cases and recovers over time using sequential Monte Carlo simulation [23]. The potential for continued human-to-human transmission of COVID-19 can be modeled by estimating the basic reproduction number [24]. The effects of air pollution-to-human transmission and human-to-human transmission on COVID-19 dynamics were investigated for epidemiological data arranged by The Ministry of Health in Italy. The data analysis was performed based on statistical methods with a multiple regression model such as the linear log-log method and the quadratic model for considering the relationship between dependent (like infected individuals) and independent (predicted from dependent) variables [25]. The effects of strict bans to prevent the transmission of COVID-19 in Italy were examined based on testing of the spatially explicit type that takes into account the spreading wave of infection leaping from the first exit point to all areas. The critical contribution of asymptomatic and presymptomatic transmission was estimated by analyzing the mobility network parameters of 107 provinces with the SEIR-like model, in which the epidemiological reporting uncertainty and the time dependence of human mobility matrices were taken into account [26].

In our recent paper, we introduced the SpID model which includes second-order suspicious $(S_{p})$, infected $(I)$, and death $(D)$ sub-models [27]. The developed SpID model is homogenous since its sub-models do not consider any external impacts such as the non-pharmacological policies. However, Suspicious-Infected-Death with Non-Pharmacological policies (SpID-N) model proposed in this paper is inhomogeneous as its sub-models consider the non-pharmacological policies (N) as external impacts. Therefore, with the SpID-N model, it is possible to incorporate the role of the non-pharmacological policies and analyze the contribution of the each policy.

Although the casualties of the outbreaks all over the world vary in terms of the numbers, peak times, and settling times; they also carry similar characters such as having a peak value and known or unknown uncertainties. Moreover, state authorities apply policies such as curfews on people having a chronic disease, age over 65, and school closures to annihilate the viruses. In this paper, without losing the generality of the developed model, we refer to COVID-19 casualties in Turkey, but since the developed SpID-N model is adaptive and the unknown parameters are learned from the available data, it can be easily modified for the casualties in other countries.

We can summarize the key contributions of the paper as

1) The SpID-N model covers the higher order internal dynamics together with the coupling dynamics,
2) The SpID-N model takes into account the multi-dimensional uncertain non-pharmacological policies,
3) The SpID-N model has completely unknown parameters and the LS-based optimization algorithm is formed to learn these unknown parameters from the available data,
4) The SpID-N model is parametric; hence, it allows extensive analysis of the future casualties and impacts of each non-pharmacological policy on the casualties.

In the rest of the paper, Section II reviews the SIR model and then introduces the proposed SpID-N model, Section III presents the parameterized models of the multi-dimensional non-pharmacological policies, Section IV formulates the LS-based parameter learning approach, Section V analysis the COVID-19 casualties in Turkey, Section VI provides the key insights of the SpID model and presents the predicted future casualties for Turkey, Section VII mentions the limitations of the study and finally, Section VIII summarizes the work.

## II. The SpID-N Model

In this section, we firstly review the SIR model, which is extensively considered for estimating casualties of the various outbreaks such as SARS and COVID-19. Then, we express the proposed SpID-N model, which is a comprehensive form of the SIR model, covering the non-pharmacological policies in an explicit way.

### A. The Sir Model

Highlighting the key properties of a well-known model plays an important role in developing a new model. Thus, in this sub-section, we provide the SIR model and its properties that we take into account in the proposed SpID-N model.

#### 1) Representation of the Sir Model

The SIR model is represented with ordinary differential equations (ODE) as

\begin{align*} \dot {S}\left ({t }\right)=&-\beta S\left ({t }\right)I(t) \\ \dot {I}\left ({t }\right)=&-\beta S\left ({t }\right)I\left ({t }\right)-\gamma R(t) \\ \dot {R}\left ({t }\right)=&\gamma R(t)\tag{1}\end{align*}

where

• $S\left ({t }\right)$ is the number of the Susceptible $(S)$ individuals,
• $I\left ({t }\right)$ is the number of the Infected $(I)$ individuals,
• $R(t)$ is the number of the Recovered $(R)$ individuals,
• $\beta $ is the transmission rate,
• $\gamma $ is the infectious rate.

#### 2) Properties of the Sir Model

The SIR model given by Equation (1) has these properties;

Property 1: It consists of the first order ordinary differential equations (ODEs), where the total order of the model is three.

Property 2: It has certain dynamics (without parametric or non-parametric random variables), since the $\beta $ and $\gamma $ parameters are known constants.

Property 3: It is homogenous (does not consider external impacts such as curfews on the weekends), as the model does not have forcing terms which can be a function of the $S$, $I,R$ or utterly independent.

Property 4: It has time-invariant dynamics since $\beta $ and $\gamma $ are constants, where these two parameters play the key roles in shaping the character of the model.

Property 5: It has slightly coupled dynamics because $S$ and $I$ ODEs feed each other, but not the $R$.

Property 6: The SIR is continuous, implying there are infinite amount of available data between two-time intervals $t$ and $t+\Delta t$, where $\Delta t$ is a small amount of time increment.

Next sub-section introduces the SpID-N model.

### B. The SpID-N Model

In this part of the paper we introduce the SpID-N model by considering the properties of the SIR model discussed in sub-section *A.*2). We can briefly summarize the key properties of the SpID-N model as 1) It takes into account the number of the suspicious $S_{p}$ casualties rather than the number of the susceptible $S$ casualties as in the SIR model since the proposed model focuses on estimating the future tests and quarantine requirements instead of the whole population, 2) It considers the death $D$ rather than the recovered $R$ casualties to use the model as the background parametric model for the artificial intelligence-based policy-making algorithm which aims to minimize a cost function consisting of the pandemic casualties, 3) Its sub-models are second-order rather than first-order since the pandemic casualties exhibit second-order properties such as the distinctive peaks in the casualties, 4) Its sub-models are discrete since the pandemic casualties are reported daily.

#### 1) SpID-N Model: Suspicious ${S}_{p}$

The suspicious $S_{p}$ part of the SpID-N model is

\begin{equation*} S_{p_{k+2}}=a_{1}S_{p_{k+1}}+a_{0}S_{p_{k}}+b_{3}I_{k}+c_{1}u_{k}\tag{2} \end{equation*}

where

• $a_{1}$ is the internal unknown parameter for the first mode of the suspicious $S_{p}$,
• $a_{0}$ is the internal unknown parameter for the second mode of the suspicious $S_{p}$,
• $b_{3}$ is the unknown coupling parameter of the infected $I$,
• $c_{1}$ is the unknown parameter of the non-pharmacological policies,
• $u_{k}$ is the sum of the multi-dimensional non- pharmacological policies,
• $k$ is the sample of discrete time (here $k$ is the days)

These unknown parameters will be learned from the available $S_{p}$, $I$, and $D$ data in Section IV. In the next sub-section, we introduce the infected part of the SpID-N model.

#### 2) SpID-N Model: Infected ${I}$

The infected $I$ part of the SpID-N model is

\begin{equation*} I_{k+2}=b_{1}I_{k+1}+b_{0}I_{k}+a_{3}S_{k}+d_{3}D_{k}+c_{2}u_{k}\tag{3} \end{equation*}

where

• $b_{1}$ is the internal unknown parameter for the first mode of the infected $I$,
• $b_{0}$ is the internal unknown parameter for the second mode of the infected $I$,
• $a_{3}$ is the unknown coupling parameter of the suspicious $S_{p}$,
• $d_{3}$ is the unknown coupling parameter of the death $D$,
• $c_{2}$ is the unknown parameter of the non-pharmacological policies $u_{k}$,

Similarly, these unknown internal, coupling, and policy parameters will be learned in Section IV. Finally, in the next sub-section, we express the death part of the SpID-N model.

#### 3) SpID-N Model: Death ${D}$

The death $D$ part of the SpID-N model is

\begin{equation*} D_{k+2}=d_{1}D_{k+1}+d_{0}D_{k}+b_{4}I_{k}+c_{3}u_{k}\tag{4} \end{equation*}

where

• $d_{1}$ is the internal unknown parameter for the first mode of the death $D$,
• $d_{0}$ is the internal unknown parameter for the second mode of the death $D$,
• $b_{4}$ is the unknown coupling parameter of the infected $I$,
• $c_{3}$ is the unknown parameter of the non-pharmacological policies $u_{k}$,

Again, these unknown internal, coupling, and policy parameters will be learned in Section IV by using the LS-based optimization algorithm. In the next section, we construct the parametrized non-pharmacological policies $u_{k}$ step-by-step.

## III. SpID-N Model: The Non-Pharmacological Policies N

In this section, we construct the multi-dimensional non-pharmacological policies step by step which can be easily modified for different cases. Parametrizing these non-pharmacological policies is necessary since their corresponding data are not directly available. Therefore, by using the well-known facts and intuitive insights about the pandemic diseases, parametric models of the non-pharmacological policies are derived by using signal processing and mathematical approaches.

### A. The Non-Pharmacological Policies

The multi-dimensional non-pharmacological model covers curfews on people 1) with chronic disease, 2) people age over 65, 3) people under age 20, 4) restrictions on weekends and holidays, 5) closure of the schools and universities.

#### 1) The Non-Pharmacological Policies: Curfews on People With Chronic Disease

**FIGURE 1.**
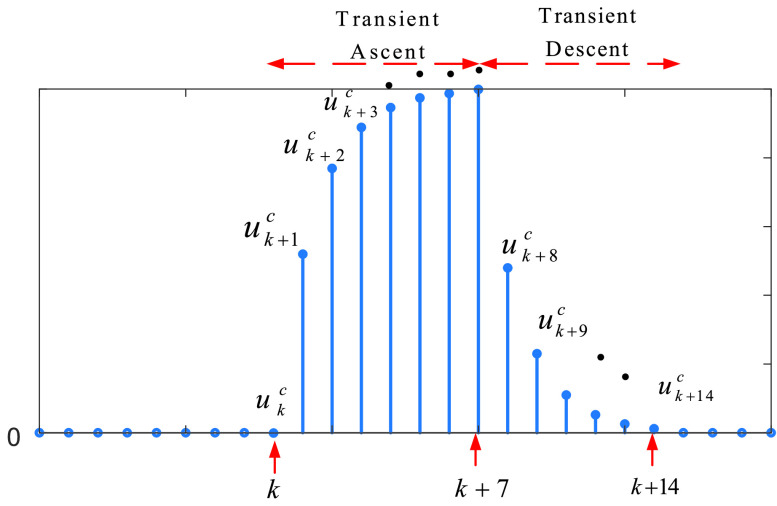
The probability distribution of appearance of the symptoms, where ${k}$ is the day that the curfew starts.

State authorities primarily focus on protecting people with chronic diseases as they are much more vulnerable to outbreaks. Therefore, curfews on them are implemented for a duration of time. Note that the symptoms of being infected can appear in 14 days where the peak point of probability occurs around day 7, which is reported by the WHO [28], as shown in Fig. 1.

As can be seen in Fig. 1, when an action is taken against the outbreak, its positive impact (response) will appear in 14 days with a possible transient ascent and a transient descent part. The transient ascent part of the response can be mathematically modelled as

\begin{equation*} u_{k}^{c}=n^{c}\left ({1-\alpha ^{k} }\right)\tag{5} \end{equation*}

where

• $u_{k}^{c}$ is the response of the curfew on the people with chronic disease,
• $n^{c}$ is the scaling factor of the number of the people with chronic disease,
• $\alpha $ is the discount factor of the impact,
• $k$ is the sample of discrete time (here $k$ is the days)
• $c$ represents chronic disease

Note that for $\alpha =0.71$ and $k=7$, the response (impact) in Fig. 1 reaches its maximum. In reality, since the response is not certain, we add random non-parametric uncertainty $\sigma ^{c}$ in Equation (5) as

\begin{equation*} u_{k}^{c}=n^{c}\left ({1-\alpha ^{k-k_{i}}+\sigma ^{c} }\right),\quad \mathrm { for}~k=k_{i},\ldots,k_{n}\tag{6} \end{equation*}

where $k_{i}$ represents the start day of the curfew, $k_{n}=k_{i}+7$ for this case. In terms of the transient descent part of the response in Fig. 1, the mathematical model is

\begin{equation*} u_{k}^{c}=n^{c}\left ({\alpha ^{k-k_{n}-1}+\sigma ^{c} }\right),\quad \mathrm { for}~k=k_{n}+1,\ldots k_{t}\tag{7} \end{equation*}

where $k_{t}=k_{n}+7$.

**FIGURE 2.**
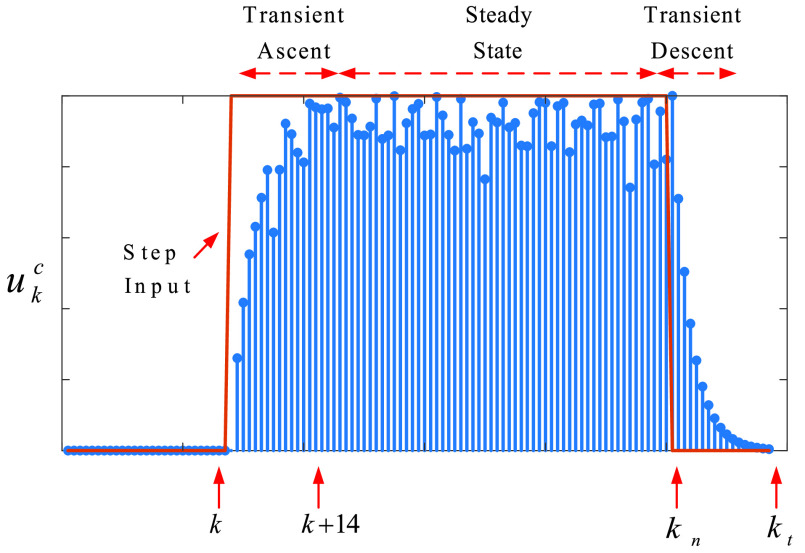
Impact of the curfew on the people with chronic disease, where ${k}$ is the day that the curfew starts.

So far in this sub-section,

• We have modelled the impacts (response) of only a one-day curfew, which has an impulse effect on fighting the COVID-19 (Fig. 1).
• However, curfews on the people with chronic disease have been implemented for a duration of time, which has a constrained step input effect on fighting the COVID-19 (Fig. 2).

In Fig. 2;

• Step input represents the duration of the curfews on people with chronic diseases.
• The impact (response) is now uncertain due to added randomness $\sigma ^{c}$ in Equations (6) and (7). This uncertainty represents the unmeasured or undetected casualties and people who violate the curfew.
• To extend the model with steady-state, modifying $k_{n}=k_{i}^{c}+k_{n}^{c}$, with $k_{i}^{c}$ is the start day and $k_{n}^{c}$ is the duration of the curfew, is enough.
• In this case, $k_{t}=k_{n}+14$.

The next sub-section presents the non-pharmacological model for the curfew imposed on people age over 65.

#### 2) The Non-Pharmacological Policies: Curfews on People Age Over 65

A further group of people prone to be infected from the outbreaks is age over 65. Thus, the state authorities implement curfews on these people primarily. The model of the curfew on the people age over 65 is closely related to the model for people with chronic diseases. Henceforth, we can slightly modify the model transient ascent and steady-state parts as

\begin{equation*} u_{k}^{65}=n^{65}\left ({1-\alpha ^{k-k_{i}^{65}}+\sigma ^{65} }\right),\quad \mathrm {for}~k=k_{i}^{65},\ldots,k_{n}\tag{8} \end{equation*}

where

• $u_{k}^{65}$ is the response of the curfew on the people age over 65,
• $n^{65}$ is the scaling factor of the number of people with age 65,
• $\alpha $ is the discount factor of the impact,
• $\sigma ^{65}$ is the random uncertainty in the response,
• $k_{n}=k_{i}^{65}+k_{n}^{65}$ where $k_{i}^{65}$ is the start day and $k_{n}^{65}$ is the duration of the curfew,
• 65 represents age over 65

Now we can present the transient descent part as

\begin{equation*} u_{k}^{65}=n^{65}\left ({\alpha ^{k-k_{n}-1}+\sigma ^{65} }\right),\quad \mathrm {for}~k=k_{n}+1,\ldots k_{t}\tag{9} \end{equation*}

where $k_{t}=k_{n}+14$. The next sub-section provides the modified model for the curfew on people age under 20.

#### 3) The Non-Pharmacological Policies: Curfews on People Age Under 20

Even though the young people are much more resistant to the outbreaks, since they spread the virus more than others, curfews on people aged under 20 have been implemented. The model of the curfew on the people age under 20 is closely affiliated with the model for the people with chronic diseases. Therefore, we can slightly modify the transient ascent and steady-state parts of the model as

\begin{equation*} u_{k}^{20}=n^{20}\left ({1-\alpha ^{k-k_{i}^{20}}+\sigma ^{20} }\right),\quad \mathrm {for}~k=k_{i}^{20},\ldots,k_{n}\tag{10} \end{equation*}

where

• $u_{k}^{20}$ is the response of the curfew on the people age under 20,
• $n^{20}$ is the scaling factor of the number of people age under 20,
• $\alpha $ is the discount factor of the impact,
• $\sigma ^{20}$ is the random uncertainty in the response,
• $k_{n}=k_{i}^{20}+k_{n}^{20}$ where $k_{i}^{20}$ is the start day and $k_{n}^{20}$ is the duration of curfew,
• 20 represents age under 20

The transient descent part as

\begin{equation*} u_{k}^{20}=n^{20}\left ({\alpha ^{k-k_{n}-1}+\sigma ^{20} }\right),\quad \mathrm {for}~k=k_{n}+1,\ldots k_{t}\tag{11} \end{equation*}

where $k_{t}=k_{n}+14$. The next sub-section expresses the model for the curfews applied on weekends and holidays.

#### 4) The Non-Pharmacological Policies: Curfews on Weekends and Holidays

Since people travel and visit each other during the weekends and holidays, viruses can spread to the mass populations. Therefore, curfews are taken into account during the weekends and holidays. Since this kind of curfews are implemented at certain intervals, it is modelled with piecewise impulses as

\begin{align*} u_{i,k}^{\mathrm {wh}}=&n^{\mathrm {wh}}\left ({1-\alpha ^{k-k_{i}^{\mathrm {wh}}}+\sigma _{i}^{\mathrm {wh}} }\right)\delta _{i}, \\&\mathrm {for}~\begin{cases} k=k_{i}^{\mathrm {wh}},\ldots,k_{n} \\ \delta _{i}=0,&\mathrm {curfew} \\ \delta _{i}=1,&\mathrm {without~curfew} \\ \end{cases}\tag{12} \end{align*}

where

• $\delta _{i}$ is the impulse representing the existence of the curfews,
• $u_{i,k}^{\mathrm {wh}}$ is the response of the curfew on the weekends and holidays,
• $n^{\mathrm {wh}}$ is the scaling factor of the number of the people under curfews on weekends and holidays,
• $\alpha $ is the discount factor of the impact,
• $\sigma _{i}^{\mathrm {wh}}$ is the random uncertainty in the response,
• $k_{n}=k_{i}^{\mathrm {wh}}+k_{n}^{\mathrm {wh}}$ where $k_{i}^{\mathrm {wh}}$ is the start day and $k_{n}^{\mathrm {wh}}=7$ is the half duration of the impact,
• wh represents weekends and holidays

The transient descent part as

\begin{align*} u_{i,k}^{\mathrm {wh}}=&n^{\mathrm {wh}}\left ({\alpha ^{k-k_{n}-1}+\sigma ^{\mathrm {wh}} }\right)\delta _{i}, \\&\mathrm {for}~\begin{cases} k=k_{n}+1,\ldots k_{t} \\ \delta _{i}=1,&\mathrm {curfew} \\ \delta _{i}=0,&\mathrm {without~curfew} \\ \end{cases}\tag{13} \end{align*}

where $k_{t}=k_{n}+14$. Fig. S2 shows the weekend and holiday curfews and their corresponding individual responses.

The total response $u_{k}^{\mathrm {wh}}$ is the sum of each response $u_{i,k}^{\mathrm {wh}}$ as

\begin{equation*} u_{k}^{\mathrm {wh}}=\sum \nolimits _{i=k-14}^{k} u_{i,k}^{\mathrm {wh}}\tag{14} \end{equation*}

**FIGURE 3.**
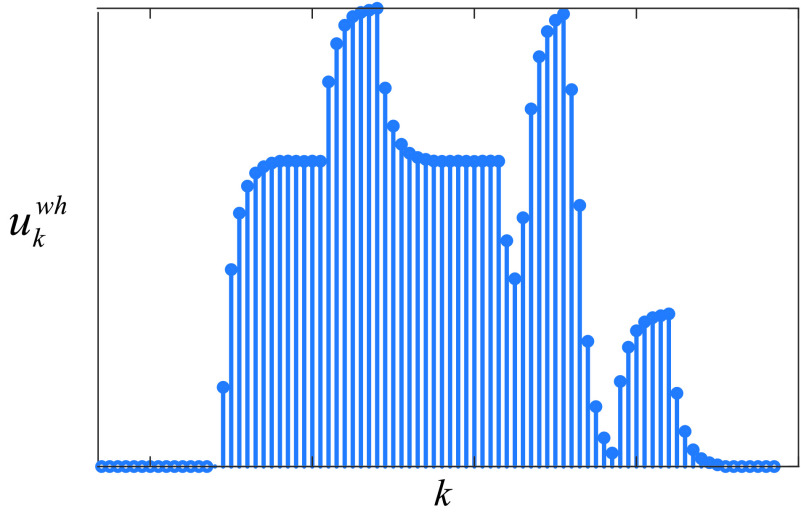
Total response of the curfews on weekends and holidays, where ${k}$ is the day that the curfew starts.

Fig. 3 shows the sum of the responses at each sample $k$.

As can be seen from Fig. 3, the response of the curfews on the weekends and holidays has multiple peaks and multiple steady-states due to their impulse type effects. The next sub-section expresses the model for the schools and universities closure.

#### 5) The Non-Pharmacological Policies: Schools and Universities Closure

As the school and universities are the mass gatherings places for the students where they actively engage with each other, the schools and universities’ closure plays a crucial role to control the spread of the virus. Since it is not a curfew, its role is mainly removing a negative effect and acting positively for a duration of time. Therefore, it has a similar modelling approach of single positive impulse as

\begin{equation*} u_{k}^{\mathrm {su}}=n^{\mathrm {su}}\left ({1-\alpha ^{k-k_{i}^{\mathrm {su}}}+\sigma ^{\mathrm {su}} }\right),\quad \mathrm {for }~k=k_{i}^{\mathrm {su}},\ldots,k_{n}\tag{15} \end{equation*}

where

• $u_{k}^{\mathrm {su}}$ is the response of the single impulse representing the schools and universities closure,
• $n^{\mathrm {su}}$ is the scaling factor of the number of students,
• $\alpha $ is the discount factor of the impact,
• $\sigma ^{\mathrm {su}}$ is the random uncertainty in the response,
• $k_{n}=k_{i}^{\mathrm {su}}+k_{n}^{\mathrm {su}}$,where $k_{i}^{\mathrm {su}}$ is the start day and $k_{n}^{\mathrm {su}}$ is the duration of the curfew,
• su represents schools and universities

The transient descent part as

\begin{equation*} u_{k}^{\mathrm {su}}=n^{\mathrm {su}}\left ({\alpha ^{k-k_{n}-1}+\sigma ^{\mathrm {su}} }\right),\quad \mathrm {for}~k=k_{n}+1,\ldots k_{t}\tag{16} \end{equation*}

where $k_{t}=k_{n}+7$. The next sub-section expresses the overall responses of the non-pharmacological policies.

### B. Total Responses of the Non-Pharmacological Policies

To form a base in Section IV, we should specify the $u_{k}$ in the sub-models given by Equations (2), (3), and (4). The total responses of all non-pharmacological policies introduced in Section III. *A*.1)–5) as

\begin{equation*} u_{k}=u_{k}^{c}+u_{k}^{65}+u_{k}^{20}+u_{k}^{\mathrm {wh}}+u_{k}^{\mathrm {su}}\tag{17} \end{equation*}

Even though they are summed up with equal importance in Equation (17), their importance is shaped by the unknown but learned $c_{1}$, $c_{2}$, and $c_{3}$ parameters in Equations (2), (3), and (4). The next sub-section expresses the key properties of the developed SpID-N model.

### C. Key Properties of the SpID-N Model

The key features of the SpID-N model are;

Property 1: Each ODE of the SpID-N model given by Equations (2), (3), and (4) is the second order. So that together with the stable and unstable modes of the COVID-19, damping and natural frequencies of the outbreak can be considered.

Property 2: The model of the non-pharmacological policies has non-parametric uncertainties $\sigma ^{c}$, $\sigma ^{65}$, $\sigma ^{20}$, $\sigma ^{\mathrm {wh}}$, and $\sigma ^{\mathrm {su}}$. So that the SpID-N model covers the random variations, too.

Property 3: The SpID-N model takes into account the non-pharmacological actions against the outbreaks such as the curfews and restrictions. Therefore, the ODEs are inhomogeneous.

Property 4: The SpID-N model does not cover the known parameters such as the transmission rate $\beta $ and infectious rate $\gamma $ which are likely to change seasonably. However, the SpID-N model assigns all the parameters as unknown and learns them from the available $S_{p}$, $I$, and $R$ data by performing an LS-based optimization.

Property 5: The number of suspicious people affects the number of infected people and also the number of recovered and dead people. Henceforth, the model is strongly coupled.

Property 6: Since it is a fact that the casualties are revealed daily, not every moment of time $\Delta t$, the SpID-N model is discrete-time instead of continuous-time.

In the next section, we provide an LS-based optimization approach to determine the unknown parameters of the proposed SpID-N model.

## IV. LS-Based Parameter Learning

This section formulates the bases and the unknown parameter vectors of the SpID-N model together with the labeled real output. This section also provides a derivation of the batch type LS-based unknown parameter estimation approach to learn the unknown parameters offline.

### A. Construction of the Bases

To learn the unknown parameters of the SpID-N model, we perform the LS-based optimization. Therefore, we create unknown functions with unknown parameters, but with the known bases, which carry crucial information about the unknown parameters. In terms of the basis of the suspicious model, consider the right-hand side of the discrete model given by Equation (2) and form the corresponding basis $\emptyset _{S_{p}}$ as

\begin{align*} \emptyset _{S_{p}}=&\left [{ S_{p}\left ({2,\ldots,N-1 }\right)S_{p}\left ({1,\ldots,N-2 }\right) }\right. \\&\left.{ I\left ({1,\ldots,N-2 }\right)u_{k}\left ({1,\ldots,N-2 }\right) }\right]^{T}\tag{18} \end{align*}

where $N$ is the length of the data. Similarly, to construct the basis for the infected $\emptyset _{I}$, consider the right-hand side of the discrete model given by Equation (3) which yields

\begin{align*} \emptyset _{I}=&\left [{ I\left ({2,\ldots,N-1 }\right)I\left ({1,\ldots,N-2 }\right)S_{p}\left ({1,\ldots,N-2 }\right) }\right. \\&\left.{ D\left ({1,\ldots,N-2 }\right)u_{k}\left ({1,\ldots,N-2 }\right) }\right]^{T}\tag{19} \end{align*}

Lastly, take into account the right-hand side of the discrete model given by Equation (4) to construct the basis for the deaths $\emptyset _{D}$ as

\begin{align*} \emptyset _{D}=&\left [{ D\left ({2,\ldots,N-1 }\right)D\left ({1,\ldots,N-2 }\right) }\right. \\&\left.{ I\left ({1,\ldots,N-2 }\right)u_{k}\left ({1,\ldots,N-2 }\right) }\right]^{T}\tag{20} \end{align*}

The bases (18), (19), and (20) are multi-dimensional and carry information about the internal dynamics of the COVID-19 and also the non-pharmacological policies. Since these bases are constructed by utilizing the facts-based insights, the unknown model parameters associated with these bases are exact as discussed next.

### B. Estimated and Parametrized Casualties

The estimated model consists of the unknown parameter vectors representing the unknown parameters of the highly coupled SpID-N model defined as

\begin{align*} w_{S_{p}}=&\left [{ a_{1}\quad {a}_{0}\quad b_{3}\quad c_{1} }\right]^{T} \\ w_{I}=&\left [{ b_{1}\quad {b}_{0}\quad a_{3}\quad {d}_{3}\quad c_{2} }\right]^{T} \\ w_{D}=&\left [{ d_{1}\quad {d}_{0}\quad b_{4}\quad c_{3} }\right]^{T}\tag{21} \end{align*}

where $w_{S_{p}}$, $w_{I}$, and $w_{D}$ are the unknown parameter vectors of the suspicious, infected, and death models respectively. The estimated individual models are

\begin{align*} \hat {y}_{S_{p}}=&w_{S_{p}}^{T}\emptyset _{S_{p}} \\ \hat {y}_{I}=&w_{I}^{T}\emptyset _{I} \\ \hat {y}_{D}=&w_{D}^{T}\emptyset _{D}\tag{22} \end{align*}

where $\hat {y}_{S_{p}}$, $\hat {y}_{I}$, and $\hat {y}_{D}$ are estimated outputs or future casualties for the suspicious, infected, and death models. To perform the LS optimization, the next step is to label the real outputs presented next.

### C. The Real Outputs

To construct the real outputs, which carry correlated information about the past casualties and non-pharmacological policies, consider the left-hand sides of the discrete models (2), (3), and (4). The real outputs (non-parametrized) are

\begin{align*} y_{S_{p}}=&S{(3,\ldots,N)}^{T} \\ y_{I}=&I{(3,\ldots,N)}^{T} \\ y_{D}=&D{(3,\ldots,N)}^{T}\tag{23} \end{align*}

where $y_{S_{p}}$, $y_{I}$, and $y_{D}$ are the real outputs. Finally, the next sub-section formulates the LS approach.

### D. LS Formulation

Consider the real outputs (23) and estimated outputs (22) by reducing the indices of the parameters and variables. The error between the real and estimated outputs reveals information about the unknown parameter vector (21). The error vector $e$ is

\begin{equation*} e=y-\hat {y}\tag{24} \end{equation*}

where $y=\left [{ y_{S_{p}}y_{I}y_{D} }\right]^{T}$ and $\hat {y}=\left [{ \hat {y}_{S_{p}}\hat {y}_{I}\hat {y}_{D} }\right]^{T}$. To ensure positive definiteness in the estimates, square the error $e$ in (24) and expand as

\begin{align*} e^{2}=&\left ({y-w^{T}\emptyset }\right)^{T}\left ({y-w^{T}\emptyset }\right) \\=&y^{T}y-w\emptyset ^{T}y-y^{T}w^{T}\emptyset +w\emptyset ^{T}w^{T}\emptyset\tag{25} \end{align*}

The slope in error determines both the direction and magnitude of the unknown parameters $w$ and moving towards the direction of the error slope minimizes the squared error (25). For the gradient descent based optimization, take the gradient of (25) as

\begin{equation*} \frac {\partial e^{2}}{\partial w}=-2\emptyset ^{T}y+2\emptyset ^{T}\emptyset w\tag{26} \end{equation*}

The unknown parameter vector $w$ setting (26) to zero is obtained as

\begin{equation*} w=\left ({\emptyset ^{T}\emptyset }\right)^{-1}\emptyset ^{T}y\tag{27} \end{equation*}

This formulation of the unknown parameter vector (27) can now be used to analyze the developed model and to predict the future casualties of the COVID-19 in Section VI.

### E. Pseudo-Code for the SpID-N Model

**Inputs:** Reported casualties $S_{p}$, $I$, and $D$ with length $N$

Constructed non-pharmacological policies $u_{k} $

Initialized unknown parameters in Equation (21)

**Outputs:** Estimated parameters of the SpID-N model

1. Construct the bases in Equations (18), (19), and (20).
2. Construct the unknown parameters in Equation (21).
3. Construct the estimated outputs in Equation (22).
4. Construct the real outputs in Equation (23).
5. Determine the unknown parameters with Equation (27).

## V. Analysis of the Data: COVID-19 Casualties in Turkey

We shortly provide and analyze the COVID-19 casualties in Turkey between 12 of March 2020 to 8 of August 2020. This section is mainly for gaining insights about the COVID-19 casualties and reflecting these insights into the comprehensive model derivation process in Sections II, III, and IV. In addition, the discussions in this section help understanding the analysis of the model and predicted future casualties in Section VI.

### A. Suspicious Casualties

**FIGURE 4.**
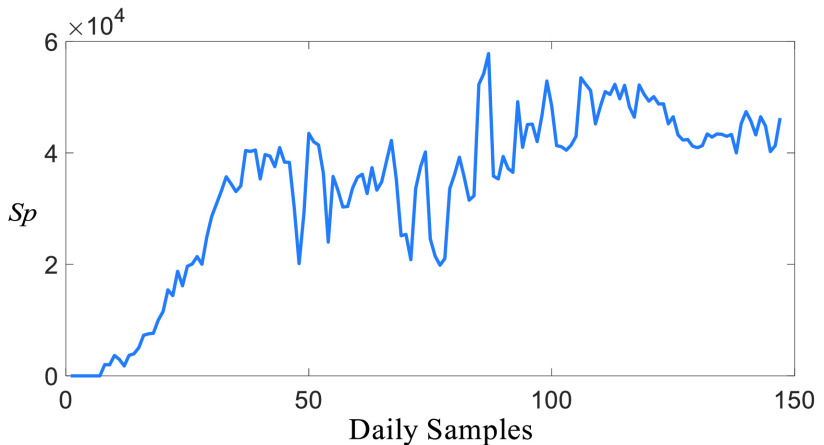
Daily suspicious casualties of Turkey, ${S}_{p}$ represents the number of the suspicious casualties.

Fig. 4 shows daily suspicious casualties, which is only the number of daily tests, reported by the Health Ministry of Turkey [29]. It can be clearly seen that the number of suspicious people has risen sharply without a distinctive peak. Even though there is a small reduction in the suspicious casualties between 40 and 70 daily samples as a result of various curfews and restrictions, it continues to be large.

**FIGURE 5.**
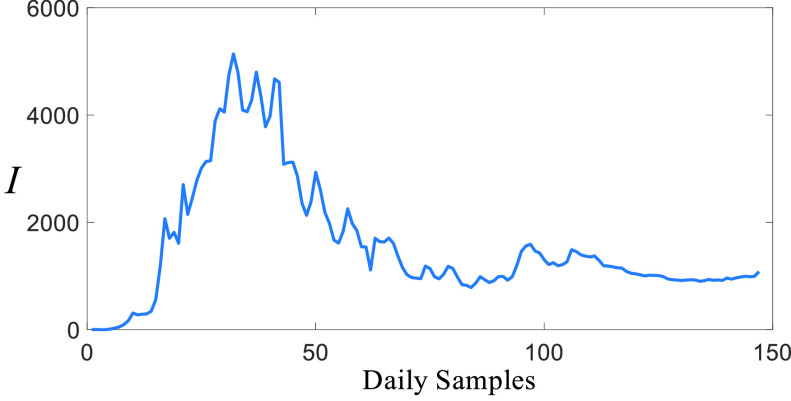
Daily infected casualties of Turkey, ${I}$ represents the number of the infected casualties.

**FIGURE 6.**
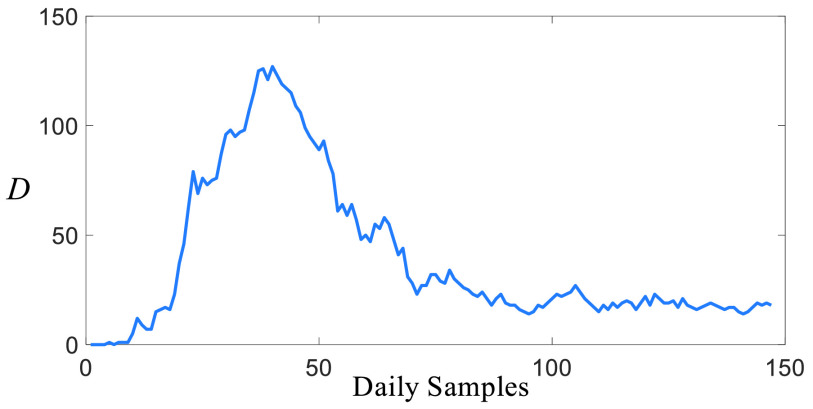
Daily death casualties of Turkey, ${D}$ represents the number of the death casualties.

However, considering the infected casualties shown in Fig. 5 and death casualties in Fig. 6, it can be deduced that the suspicious casualties are mostly for searching people who might be infected or taking pre-cautious actions to prevent people such as the military stuff. It is expected that this fact reduces the coupling effect among the suspicious, infected, and death casualties.

### B. Infected Casualties

Fig. 5 shows daily infected casualties reported by the Health Ministry of Turkey [29]. Fig. 5 also shows a distinct and sharp peak with random oscillations due to uncertainties. This peak implies the second-order dynamics discussed in Sections II and III. It is also noticeable that after the peak, infected casualties settle down a region as a result of the impacts of the non-pharmacological policies. However, the infected casualties do not converge zero and they fluctuate around a bounded equilibrium. This is highly likely due to removing all the restrictions and curfews on 1 of June. This has caused improving uncertainty as well.

### C. Death Casualties

Fig. 6 shows the number of deaths stemmed from the COVID-19 reported by the Health Ministry of Turkey [29]. It is clear that the number of deaths (Fig. 6) and the number of infected people (Fig. 5) have a significantly similar character as opposed to the number of suspicious people (Fig. 4). It is clear that the number of deaths has reduced from 130s to 20s, but it fluctuates around a non-zero equilibrium point.

## VI. Analysis of the SpID-N Model

This section provides an insightful analysis of the coupled and higher-order parametric SpID-N model.

### A. Parameters of the SpID-N Model

We provide parameters of the SpID-N model for the case of casualties in Turkey to make insightful comments about the COVID-19 analysis. However, since the developed model is flexible, providing corresponding parameters yields a specific model for that country.

Based on the knowledge given by Table 1 [30], we can now specify the corresponding SpID-N parameters in Table 2 [30].

**TABLE 1:** Population Characteristics of Turkey [30]

Total population	83.154.000
People with chronic diseases	26.567.000
People age over 65	7.550.000
People age over 65 with chronic diseases	6.032.000
People age over 65 without chronic diseases	1.517.000
People age under 20	25.573.000
Remaining people under curfews	29.497.000
Number of the school and university students	26.048.000

**TABLE 2:** Parameters of the SpID-N Model [30]

Symbol	Values	Explanation
$n^{c}$	26.567.000	People with chronic diseases
$n^{65}$	1.517.000	People age over 65 without chronic diseases
$n^{20}$	25.573.000	People age under 20
$n^{\textrm {wh}}$	29.497.000	Remaining people under curfews
$n^{\textrm {su}}$	26.048.000	Number of the school and university students
$k_{i} $	23/02/$2020\,\,k_{i} =1$	First COVID-19 casualty has seen
$k_{i}^{c} $ and $k_{n}^{c} $	21/03/2020–01/06/2020 $k_{i}^{c} =28$ $k_{n}^{c} =100$	Start and end dates of the curfews for the people with chronic diseases and their corresponding day numbers
$k_{i}^{65} $ and $k_{n}^{65} $	21/03/2020–01/06/2020 $k_{i}^{65} =28$ $k_{n}^{65} =100$	Start and end dates of the curfews for the people age over 65 and their corresponding day numbers
$k_{i}^{20} $ and $k_{n}^{20} $	03/04/2020–01/06/2020 $k_{i}^{20} =41$ $k_{n}^{20} =100$	Start and end dates of the curfews for the people age under 20 and their corresponding day numbers
$k_{i}^{\textrm {su}} $ and $k_{n}^{\textrm {su}} $	16/03/2020 – cont. $k_{i}^{\textrm {su}} =23$ $k_{n}^{\textrm {su}} =24$	Start and end dates of the schools and universities closures and their corresponding day numbers
$k_{i}^{\textrm {wh}} $ and $k_{n}^{\textrm {wh}} $	10/04/2020–12/04/2020 $k_{i}^{\textrm {wh}} =48$ $k_{n}^{\textrm {wh}} =49$ 17/04/2020–19/04/2020 $k_{i}^{\textrm {wh}} =55$ $k_{n}^{\textrm {wh}} =56$ 23/04/2020–26/04/2020 $k_{i}^{\textrm {wh}} =61$ $k_{n}^{\textrm {wh}} =63$ 01/05/2020–03/05/2020 $k_{i}^{\textrm {wh}} =69$ $k_{n}^{\textrm {wh}} =70$ 08/05/2020–10/05/2020 $k_{i}^{\textrm {wh}} =76$ $k_{n}^{\textrm {wh}} =77$ 16/05/2020–19/05/2020 $k_{i}^{\textrm {wh}} =84$ $k_{n}^{\textrm {wh}} =86$ 29/05/2020–31/05/2020 $k_{i}^{\textrm {wh}} =97$ $k_{n}^{\textrm {wh}} =98$	Start and end dates of the curfews for everyone and their corresponding day numbers

### B. Comments on the SpID-N Model

The learned parameters of the SpID-N model with the LS estimator (27) are

\begin{align*} S_{p_{k+2}}=&0.9453S_{k+1}+0.0047S_{p_{k}}+0.5794I_{k}+0.0153u_{k} \\ I_{k+2}=&1.0545I_{k+1}-0.0443I_{k}-0.0004S_{p_{k}} \\&-0.5334D_{k}-0.0001u_{k} \\ D_{k+2}=&1.0465D_{k+1}-0.1576D_{k}+0.0033I_{k}+0.00002u_{k}\tag{28} \end{align*}

Insight 1: Correlation among the internal effects of the $S_{p}$, $I$, $D$ casualties get stronger from $S_{p}$ to $I$ and $D$, respectively. This can be seen from the $0.0047S_{p_{k}}$, $0.0443I_{k}$, and $0.1576D_{k}$ which grow 10 folds of each other. This confirms the consistency of the infected $I$ and death $D$ data, but not the suspicious $S_{p}$ data since it covers the tests performed without strong suspicions.
Insight 2: The infected number of people has quite strong effects on the number of suspicious people due to $0.5794I_{k}$ in (28).
Insight 3: However, the role of the number of the suspicious people on the number of the infected people is limited $(0.0004S_{p_{k}})$ due to widely performed precautious tests for the people who start their duties (i.e. soldiers, workers).
Insight 4: In terms of non-pharmacological policies $u_{k}$, it plays an important role in all the casualties. Note that even though it has small coefficients such as $0.0153u_{k}$, $0.0001u_{k}$, $0.00002u_{k}$ since the $u_{k}$ have large bases weighted with the corresponding populations, its impact is significant.

### C. Eigenvalue Based Analysis of the SpID-N Model

**FIGURE 7.**
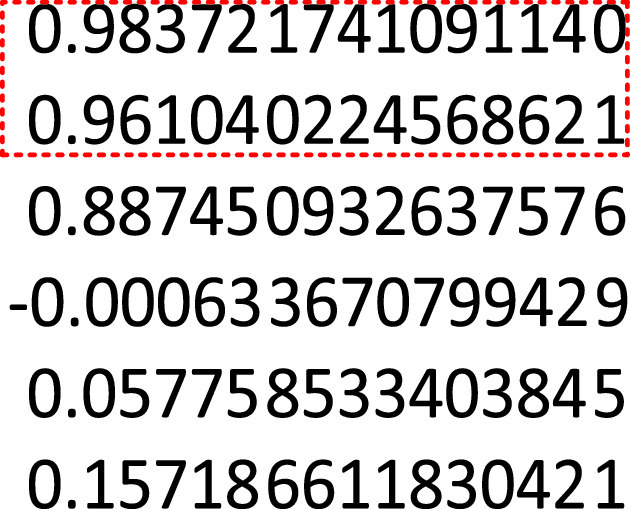
Eigenvalues of the discrete SpID-N model.

Eigenvalues of the coupled and *6*th order discrete model (28) can be used to reveal important knowledge about the future behavior of the COVID-19 casualties (decrease or increase unboundedly and the time to reach a certain level). Therefore, in Fig. 7 we provide the eigenvalues of the model without the external non-pharmacological effects.

As the model is discrete, any eigenvalue larger than unity leads all coupled outputs to blow up. Thus, all the eigenvalues of the model must be inside the unit circle for convergent outputs. As can be seen from Fig. 7, all the eigenvalues of the SpID-N model are inside the unit circle. However, two of the eigenvalues inside the dashed rectangle are close to 1; henceforth, with slight incremental changes either in the internal dynamics or external effects, the eigenvalues might move towards the unstable region. In this case, if no non-pharmacological actions are taken, the casualties increase unboundedly.

### D. Estimated Future COVID-19 Casualties

**FIGURE 8.**
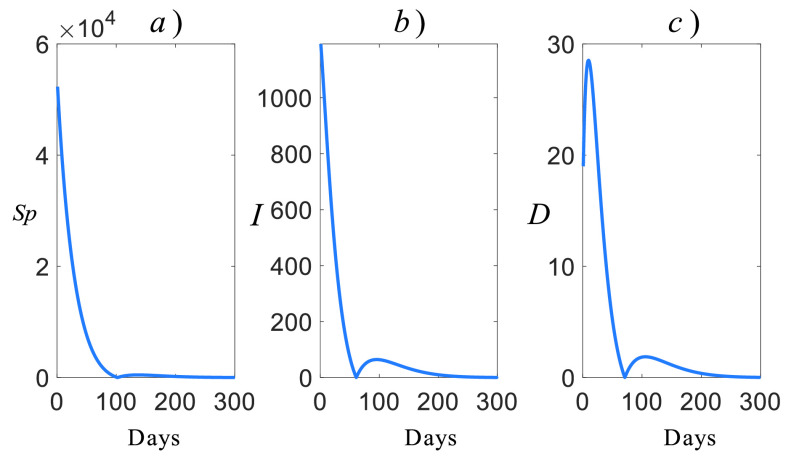
Estimated future COVID-19 casualties for Turkey, a) suspicious, b) infected, c) death.

We provide the estimated future COVID-19 casualties in Fig. 8 for Turkey by using the model given by Equation (28).

It is clear that all the $S_{p}$, $I$, and $D$ casualties decrease to zero around 100 days, but they jump back. This reveals two properties of the COVID-19:

1) The natural frequency of the model is large: This implies that the rise time of the virus is small and also it has an aggressive response.
2) The damping factor of the model is small: This implies that the lately removed restrictions and curfews have reduced the damping factor of the casualties. If no non-pharmacological policies are imposed, then fluctuations in casualties are expected.

**FIGURE 9.**
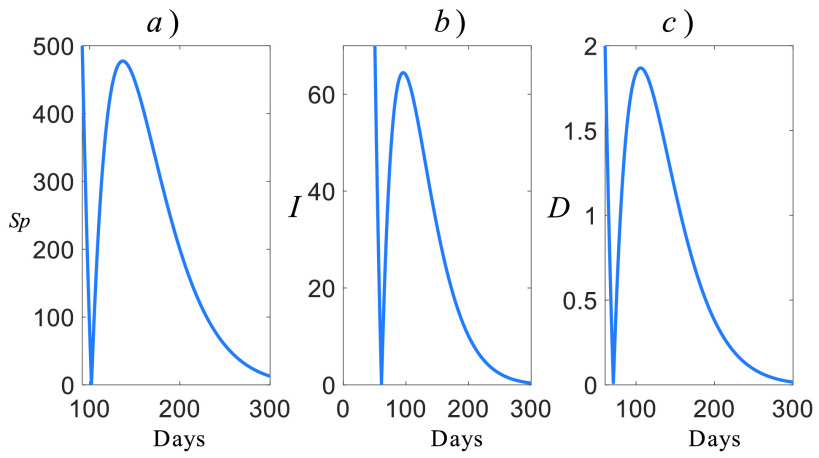
Expanded future casualties, a) suspicious, b) infected, c) deaths.

To clearly see the convergent regions of Fig. 8, we provide its expanded form around the convergent regions in Fig. 9. It can be seen that the suspicious casualties reach zero at day 100, but it jumps back to 480s casualties (Fig. 9a). In 300 days, it reduces to 30s and its slope information confirms the convergent behavior of the future suspicious casualties in the further days. In terms of the infected casualties, it hits zero around day 70 and rises back to 62 casualties (Fig. 9b). Similarly, the death casualties reach zero around day 60 and rise back around 2 casualties (Fig. 9c). Both the infected and death casualties seem to converge zero around 300 days under the current conditions.

Next sub-section analyzes the impacts of the individual non-pharmacological policies on the future estimates of the COVID-19.

### E. Analysis of the Non-Pharmacological Policies

**FIGURE 10.**
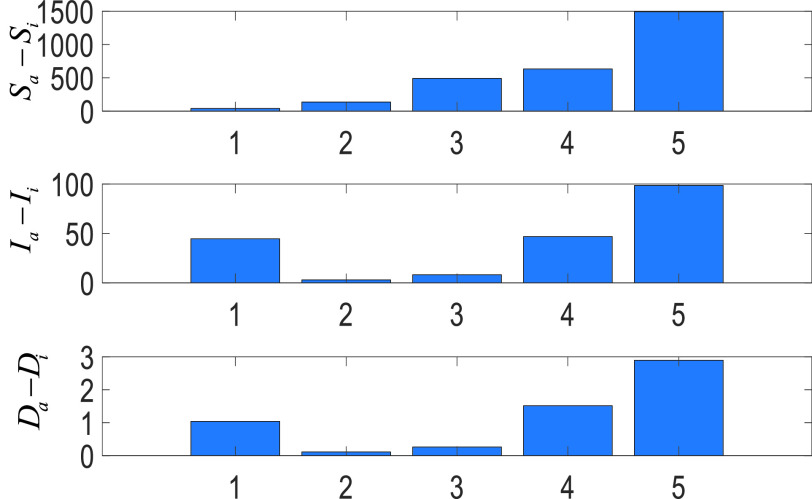
Average differences for the future COVID-19 casualties without individual non-pharmocological policies. 1) with chronic disease, 2) age over 65, 3) under age 20, 4) during the weekends and holidays, 5) impacts of closures of the schools and universities.

Fig. 10 shows differences in casualties without an individual non-pharmacological policy where the numbers represent the curfews on people

1) with chronic disease,
2) age over 65,
3) under age 20,
4) during the weekends and holidays,
5) impacts of closures of the schools and universities

and

• $S_{a}$ represents the average impacts of all non-pharmacological policies on the suspicious casualties,
• $S_{i}$ represents the impact of the non-pharmacological policies on the suspicious casualties without the $i$th non-pharmacological policy,
• $I_{a}$ represents the average impacts of all non-pharmacological policies on the infected casualties,
• $I_{i}$ represents the impact of the non-pharmacological policies on the infected casualties without the $i$th non-pharmacological policy,
• $D_{a}$ represents the average impacts of all non-pharmacological policies on the death casualties,
• $D_{i}$ represents the impact of the non-pharmacological policies on the death casualties without the $i$th non-pharmacological policy,

As can be seen from Fig. 10,

1) *Without the curfews imposed on the people with chronic disease:* Although its contribution to the number of suspicious casualties is significantly limited, its impacts on the number of the infected and also deaths casualties are significant. This shows that people with chronic diseases are more vulnerable to COVID-19 (number 1).
2) *Without the curfews imposed on the people age over 65 without a chronic disease:* Despite their moderate role in the number of suspicious casualties, their impacts on the infected and death casualties are not strong. This is because of the population of the people age over 65 (1.517.000 over 83.000.000 population as presented in Table 1) (number 2).
3) *Without the curfews imposed on the people age under 20:* It is clear that the young people cause a considerable increment in suspicious casualties. However, since they are less prone to be infected, its impacts on infectious and deaths are insignificant (number 3).
4) *Without the curfews imposed on the weekends and holidays:* Since this type of restriction is for everyone, its role on all the casualties is consistent (number 4).
5) *Without the closures of the schools and universities:* Its impacts are consistent on all the casualties due to two reasons: a) as can be seen from Table 1, almost 1/3 of the Turkish population is student, b) as can be seen in Table 2, when the schools and universities were closed, it was the only non-pharmacological policy (number 5).

## VII. Limitations of the Study

In the proposed model, the suspicious, infected, death casualties, and non-pharmacological policies were taken into consideration, however, the intensive care and intubation casualties, pharmacological policies, and unknown uncertainties were not taken into account. In addition to this, the study does not focus on the risks of infections depends on some factors such as the environmental effects, the demography of cities, and the mobility of nations.

## VIII. Conclusion

In this paper, we have proposed the SpID-N model to predict and analyze the future COVID-19 casualties. Firstly, we provide an insightful analysis of the well-known SIR model and we have determined the internal and coupled structure of the SpID-N model. Secondly, we have derived the mathematical models of the non-pharmacological policies and represented them in terms of known bases and their unknown contributions to each part of the SpID-N model. Thirdly, we formulate the LS-based optimization approach to obtain the unknown parameters of the SpID-N model. The predicted model parameters confirm that the COVID-19 casualties in Turkey are inside the stable region, but two of the modes are close to the instability region. The model also provides that the number of infected and death casualties will converge zero in 300 days, whereas the number of suspicious casualties will require more time. In addition, we have analyzed the impacts of the individual non-pharmacological policies and showed that people with chronic diseases are considerably prone to be infected and even deaths. However, even though young people spread the virus, the number of infected and death casualties among young people is low.

Thus, by using our developed and proposed SpID-N model, authorities of the countries can plan new measures against the virus in the short-medium-long term, and accordingly, update their regulations in the fields of economy, travel, and health systems according to the data analysis of the model we propose.

In our future studies, it will be possible to develop new versions that take the problems mentioned in the section of the limitations of the study into account in the model, according to our current proposed approach. Models to be developed should be combined with artificial intelligence approaches in order to determine policies for other future pandemics that humanity may encounter.
